# Purification, Preliminary Characterization and Hepatoprotective Effects of Polysaccharides from Dandelion Root

**DOI:** 10.3390/molecules22091409

**Published:** 2017-08-25

**Authors:** Liangliang Cai, Dongwei Wan, Fanglian Yi, Libiao Luan

**Affiliations:** Department of Pharmaceutics, China Pharmaceutical University, Nanjing 210009, China; cailiangliang10@163.com (L.C.); hicpu20@126.com (D.W.); wxlife_2009@163.com (F.Y.)

**Keywords:** dandelion root, polysaccharides, purification, hepatoprotective effects, Nrf2-Keap1

## Abstract

In this study, purification, preliminary characterization and hepatoprotective effects of water-soluble polysaccharides from dandelion root (DRP) were investigated. Two polysaccharides, DRP1 and DRP2, were isolated from DRP. The two polysaccharides were α-type polysaccharides and didn’t contain protein. DRP1, with a molecular weight of 5695 Da, was composed of glucose, galactose and arabinose, whereas DRP2, with molecular weight of 8882 Da, was composed of rhamnose, galacturonic acid, glucose, galactose and arabinose. The backbone of DRP1 was mainly composed of (1→6)-linked-α-d-Glc and (1→3,4)-linked-α-d-Glc. DRP2 was mainly composed of (1→)-linked-α-d-Ara and (1→)-linked-α-d-Glc. A proof-of-concept study was performed to assess the therapeutic potential of DRP1 and DRP2 in a mouse model that mimics acetaminophen (APAP) -induced liver injury (AILI) in humans. The present study shows DRP1 and DRP2 could protect the liver from APAP-induced hepatic injury by activating the Nrf2-Keap1 pathway. These conclusions demonstrate that the DRP1 and DRP2 might be suitable as functional foods and natural drugs in preventing APAP-induced liver injury.

## 1. Introduction

Acetaminophen (APAP), a commonly used over-the-counter antipyretic and analgesic, is safe in therapeutic doses, overdoses may cause acute liver injury. At therapeutic doses, most APAP is metabolized by sulfation and glucuronidation, and only a small fraction is oxidized by the cytochrome P450 system, resulting in the formation of a highly reactive intermediate metabolite called *N*-acetyl-*p*-benzoquinoneimine (NAPQI), which under normal conditions is usually detoxified via conjugation with glutathione (GSH). However, when over-dosed, NAPQI deplete hepatic glutathione and bind covalently to intracellular proteins, resulting in increased oxidative stress and hepatic necrosis [1,2].

In response to oxidative stress, cytoprotective enzymes were activated in the liver mounts. One major example of cellular defense is activation of nuclear factor erythroid-2-related factor 2 (Nrf2), which is a key factor that plays a central role in cellular defense against oxidative and electrophilic insults. Nrf2 is a transcription factor that regulates the expression of various cytoprotective enzymes by binding to the antioxidant response element (ARE) domain upstream of their promoter. Under normal conditions, Nrf2 is kept in the cytoplasm by Kelch-like epichlorohydrin-associated protein 1 (Keap1), a negative regulator of Nrf2 [3]. During oxidative stress, Nrf2 dissociates from Keap1 and translocates into the nucleus to stimulate transcription of target genes with the help of small Maf proteins [4,5]. Nrf2 target genes include glutamate-cysteine ligase catalytic subunit (GCLC), NAD(P)H: quinine oxidoreductase 1 (NQO1), and hemeoxygenase-1 (HO-1), among others. Nrf2 is assumed to be a potential target for the treatment of drug-induced toxicity. As is reported, Nrf2 knockout mice were highly susceptible to APAP-induced liver injury [6,7,8,9]. In contrast, mice with a hepatocyte-specific deletion of Keap1 exhibit an increase in Nrf2 and its target genes, resulted in a resistant to APAP-induced hepatotoxicity [10]. Because the hepatotoxicity of APAP is primarily due to oxidative stress and the Nrf2-Keap1 system plays an important role in protecting against oxidative stress, the medicine that could activate Nrf2 might be beneficial in protecting against APAP-induced liver injury.

Dandelion (*Taraxacum officinale*), a member of the Asteraceae plant family, is a perennial herb native to the Northern Hemisphere [11,12]. In China, dandelion is not only a delicious food but also a traditional and herbal medicine, used for its choleretic, diuretic, anti-rheumatic, anti-diabetic and anti-inflammatory properties [13]. Some studies have demonstrated that the extracts from different parts of the dandelion have multiple pharmacological effects. For example, the aqueous extract from dandelion roots reduced alcohol-induced oxidative stress [13]; dandelion leaf extract alleviated high-fat diet-induced nonalcoholic fatty liver [14]; and dandelion flower extract scavenged reactive oxygen species and protected DNA from reactive oxygen species (ROS)-induced damage in vitro [15]. Furthermore, dandelion polysaccharides were reported to display anti-oxidative and anti-inflammatory activities [16,17]. However, scantly data are available regarding the possible functions of the polysaccharides in dandelion root.

Recently, polysaccharides from plants and fungi have attracted increasing attention due to their medicinal values, such as anti-tumor [18,19], anti-oxidant [20], anti-diabetic [21], anti-coagulation [22], anti-injury [23] and an human immunity-enhancing properties [24]. In addition, most of them have been proved to be natural and nontoxic, ideal for producing healthcare foods or medicines [25,26]. Taken together, it is seemed necessary and significative to explore the hepatoprotective effects of the polysaccharides extracted from the dandelion root in preventing the APAP-induced liver injury.

Therefore, in this study, two homogeneous polysaccharides were isolated and purified from dandelion root by ethanol precipitation and column chromatography separation. The monosaccharide composition, molecular weight, ultraviolet (UV), Fourier transform infrared (FT-IR), gas chromatography and mass spectrometry (GC-MS) analysis revealed their preliminary characteristics. Furthermore, the protective effects of polysaccharide fractions against APAP-induced hepatotoxicity were also investigated.

## 2. Results

### 2.1. Separation and Purification of Polysaccharide

**Figure 1:**
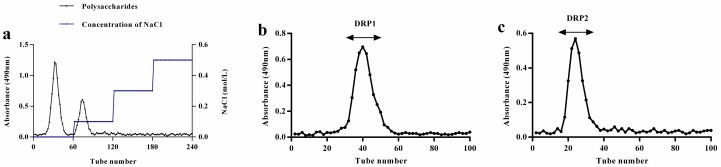
Anion-exchange chromatogram of the crude DRP on a DEAE-52 cellulose column (**a**), and gel filtration chromatograms of DRP1 (**b**) and DRP2 (**c**) on Sephacryl^TM^ S-200 column.

The crude dandelion root polysaccharide (DRP) was obtained by hot-water extraction, ethanol precipitation, and deproteinization. To purify the polysaccharide, the crude DRP was separated on a DEAE-52 cellulose column eluting with distilled water and stepwise addition of different NaCl solutions (0.1, 0.3 and 0.5 mol/L). Two polysaccharides were separated and designated as DRP1 and DRP2. DRP1 and DRP2 accounted for 70.4% and 22.8% of the total DRP content, respectively. The overall recovery was 93.2%, which revealed that the polysaccharides had been effectively eluted from the column. The DEAE-52 cellulose column chromatogram of DRP is shown in Figure 1a. Then the DRP1 and DRP2 were further purified through a Sephacryl^TM^ S-200 gel filtration column. Each polysaccharide produces only a single symmetrically sharp peak (Figure 1b,c), suggesting that their purity is very high. The DRP1 and DRP2 fractions were collected for the subsequent analysis.

### 2.2. Preliminary Characterization of Polysaccharide Fractions

#### 2.2.1. Analysis of Polysaccharide and Protein Contents

The carbohydrate contents of DRP1 and DRP2 were 98.5% and 96.1% by the phenol-sulfuric acid method. DRP1 and DRP2 had no response to the Commassie Brilliant Blue G-250 method. Neither did they have absorption at 280 nm and 260 nm in the UV scanning spectrum at 200–400 nm, indicating the absence of protein.

#### 2.2.2. FT-IR Spectroscopy

**Figure 2:**
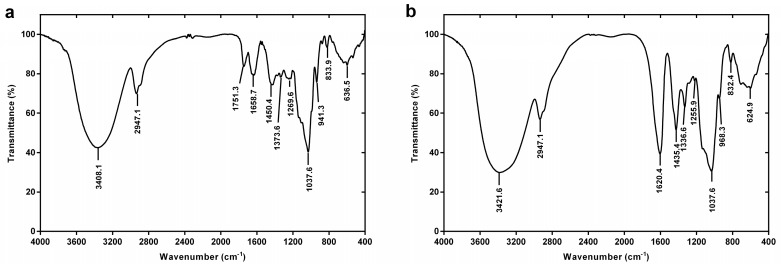
FT-IR spectra: (**a**) DRP1, (**b**) DRP2.

The Fourier transform infrared (FT-IR) spectra of DRP1 and DRP2 are shown in Figure 2. The absorption bands within the range of 3600–3000, 3000–2800, 1400–1200 and 1200–700 cm^−1^ are characteristic absorption peaks of polysaccharides. The strong and broad absorption peaks at 3408.1/3421.6 cm^−1^ were attributed to the stretching vibration of O-H which indicates hydroxyl groups exist in DRP1 and DRP2. The absorption peaks at 2947.1/2947.1 cm^−1^ were due to the stretching vibration of C-H in DRP1 and DRP2. The strong absorption peaks at 1658.7/1620.4 cm^−1^ were caused by the stretching vibration of a free carboxylic carbonyl group. The absorptions at 1450.4/1435.4 cm^−1^ in DRP1and DRP2 represent CH_2_ and OH bonding. The peaks in the range of 1300–1000 cm^−1^ were characteristic of carbohydrates. Moreover, 833.9/832.4 cm^−1^ in DRP1 and DRP2 show that the sugar linkage types were α-type glycosidic linkages [27]. These results indicated that DRP1 and DRP2 possessed typical absorption peaks of polysaccharides.

#### 2.2.3. Monosaccharide Composition Analysis

**Figure 3:**
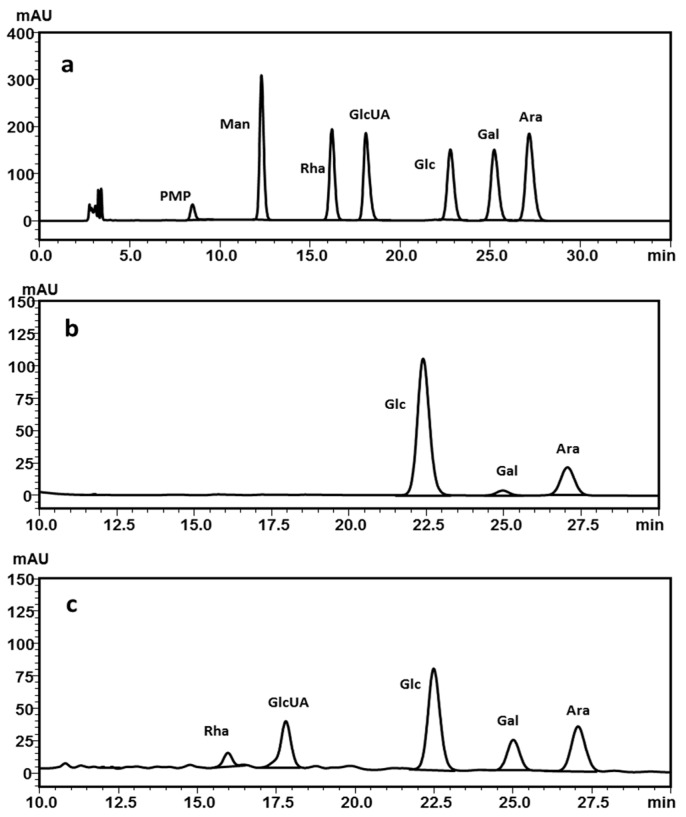
Chromatograms of monosaccharide compositions: (**a**) Six monosaccharides, (**b**) DRP1, (**c**) DRP2. PMP: 1-phenyl-3-methyl-5-pyrazolone.

In order to detect the monosaccharide components of the polysaccharides, DRP1 and DRP2 were hydrolyzed with trifluoroacetic acid, and the hydrolysate was neutralized. Then the released monosaccharide derivatives were analyzed by high performance liquid chromatography (HPLC). As identified by comparing the retention time of standards (Figure 3a), the liquid chromatography analysis showed that DRP1 was composed of glucose, galactose and arabinose, with relative molar percentages of 78.11%, 3.07% and 18.82%, respectively (Figure 3b), whereas DRP2 consisted of rhamnose, glucuronic acid, glucose, galactose and arabinose, with relative molar percentages of 4.40%, 17.84%, 42.59%, 13.34% and 21.84%, respectively (Figure 3c).

#### 2.2.4. Molecular Weight Analysis by Gel Permeation Chromatography

The weight average molecular weight (Mw) of polysaccharides is another important parameter influencing their bioactivities. High performance gel permeation chromatography (HPGPC) was applied to determine the Mw of the polysaccharide fractions. On the basis of a calibration with standard dextran, HPGPC results showed that the molecular weights of DRP1 and DRP2 were estimated to be 5695 and 8882 Da, respectively.

#### 2.2.5. Methylation and GC-MS Analysis

**Figure 4:**
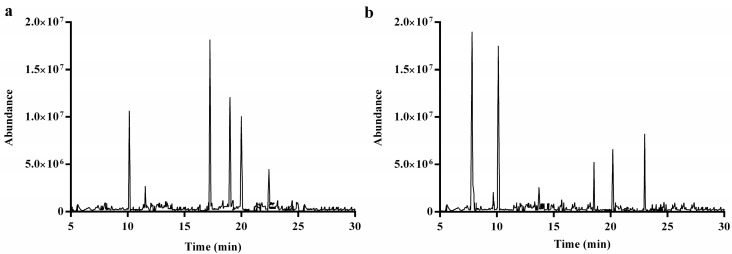
Total ion chromatograms (TICs) of DRP1 (**a**) and DRP2 (**b**).

To determine the glycosyl-linkages, DRP1 and DRP2 were methylated, hydrolyzed and converted into alditol acetates for GC-MS analysis. The total ion chromatograms (TICs) of DRP1 and DRP2 are shown in Figure 4.

The identification and the proportions of the methylated alditol acetates of DRP1 and DRP2 are given in Table 1 and Table 2. The DRP1 results showed that molar ratios of 3,5-Me_2_-Ara, 2,3,4,6-Me_4_-Gal, 2,3,4-Me_3_-Glc, 2,6-Me_2_-Glc, 3,6-Me_2_-Glc and 3,4-Me_2_-Glc were 8.95, 2.15, 15.48, 10.43, 8.67 and 3.86 according to the peak areas. Glucose-based sugar residues (including 1,6-linked Glc, 1,3,4-linked Glc, 1,2,4-linked Glc and 1,2,6-linked Glc) were highly enriched in DRP1, which was consistent with monosaccharide composition analysis. However, the DRP2 results showed that the molar ratios of 2,3,5-Me_3_-Ara, 3,4-Me_2_-Rha, 2,4-Me_2_-Glc, 2,3,4,6-Me_4_-Glc, 3,6-Me_2_-Glc, 2,4,6-Me_3_-Gal and 3,4-Me_2_-Gal were 4.96, 0.34, 1.71, 4.58, 1.37, 0.52 and 2.15 according to the peak areas.

### 2.3. Polysaccharide Fractions Protect against APAP-Induced Acute Liver Injury in Mice

#### 2.3.1. Polysaccharide Fractions Alleviated APAP-Induced Histology Changes in Liver

**Figure 5:**
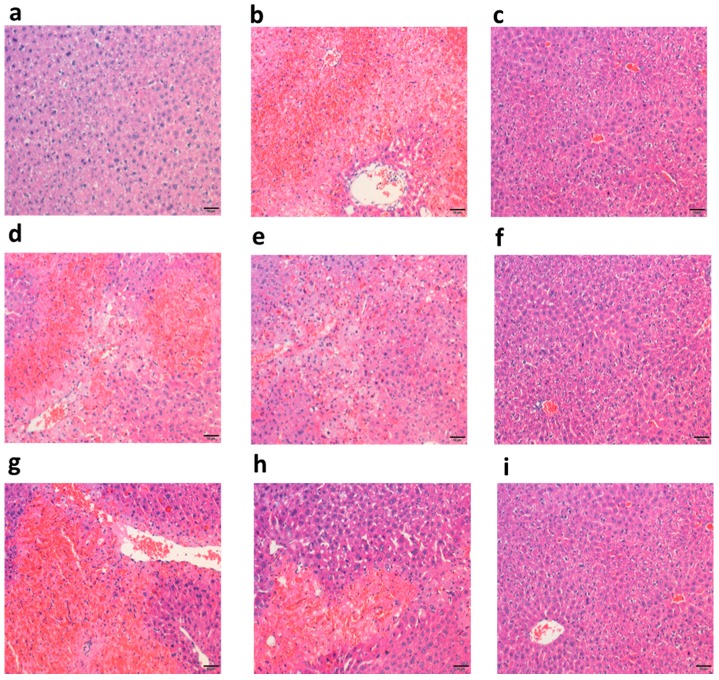
Effects of DRP1 and DRP2 on histopathological changes in liver tissues (magnification 200×). (**a**) liver of mice in the NC group; (**b**) liver of mice in the model control (MC) group; (**c**) liver of mice in the positive control (PC) group; (**d**–**f**) liver of mice injected with DRP1 at dosage of 50, 100 and 200 mg/kg; and (**g**–**h**) liver of mice injected with DRP2 at dosage of 50, 100 and 200 mg/kg, respectively.

A liver histology study was used to determine the protective effect of DRP1 and DRP2 on APAP-induced liver injury. As shown in Figure 5, liver tissues of the normal control (NC) group (Figure 5a) showed the normal liver structure. APAP treatment caused several visible histological liver changes, including extensive hemorrhages, necrosis and neutrophil infiltration (Figure 5b). However, liver from DRP1 and DRP2 treated group exhibited a significant alleviation of the liver injuries (Figure 5d–f). Meanwhile, the *N*-acetylcysteine treatment had a similar restorative effect on the APAP-induced pathological changes (Figure 5c).

#### 2.3.2. Effects of Polysaccharide Fractions on APAP-Induced Serum Aspartate Aminotransferases (AST) Levels

**Figure 6:**
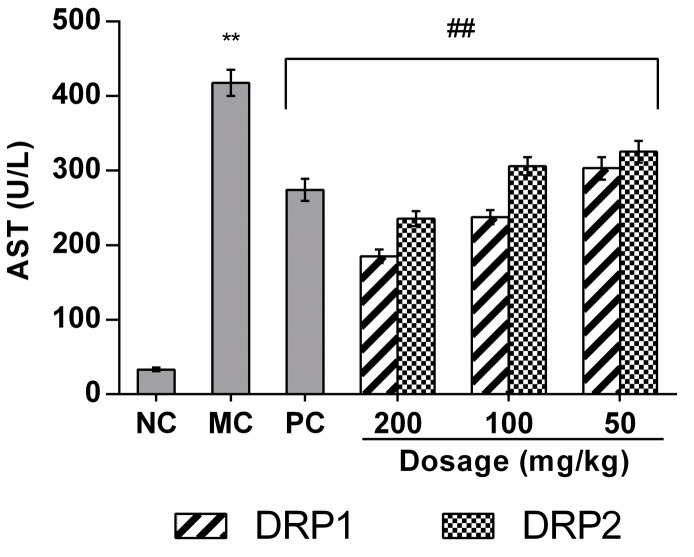
Effects of DRP1 and DRP2 on serum AST levels. The values presented are the mean ± S.E.M. (n = 10 in each group). ** *p* < 0.01 compared with NC group; ## *p* < 0.01 compared with the MC group.

Serum levels of AST are standard parameters for evaluating liver function. As shown in Figure 6, the MC group showed significantly increased serum AST levels compared with the NC group (*p* < 0.01), and DRP1 and DRP2 treatment significantly prevented the increases of these serum enzyme levels compared with the MC group (*p* < 0.01). These data indicated that after induction of APAP hepatotoxicity, treatment with DRP1 and DRP2 significantly reduces liver injury.

#### 2.3.3. Polysaccharide Fractions Inhibited APAP-Induced Oxidative Stress in Liver

**Figure 7:**
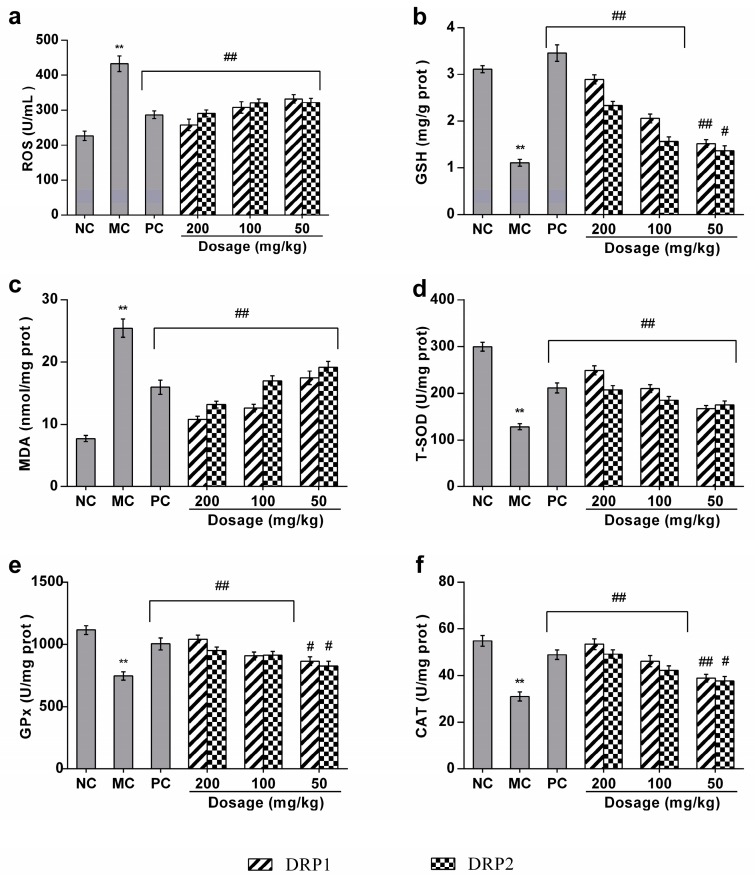
Effects of DRP1 and DRP2 on liver ROS, GSH, MDA, T-SOD, GPx and CAT levels. (**a**), ROS (**b**), GSH (**c**), MDA (**d**), T-SOD (**e**), GPx (**f**), CAT. The values presented are the mean ± S.E.M. (*n* = 10 in each group). ** *p* < 0.01 compared with the NC group; # *p* < 0.05, ## *p* < 0.01 compared with the MC group.

Many studies have suggested that the levels of reactive oxygen species (ROS), malondialdehyde (MDA) and glutathione (GSH) might be indicators of oxidative stress. As shown in Figure 7a,c, compared to the NC group, liver MDA and ROS levels were increased significantly in the APAP-treated group (*p* < 0.01). However, DRP1 and DRP2 dose-dependently inhibited APAP-induced liver ROS and MDA levels (*p* < 0.01). As shown in Figure 7b, the GSH level in APAP-treated mice was markedly decreased compared with that of the NC group (*p* < 0.01). However, treatment with DRP1 and DRP1 in APAP-treated mice significantly increased the hepatic GSH level in a dose-dependent manner (*p* < 0.01).

#### 2.3.4. Polysaccharide Fractions Reversed the Antioxidant Enzyme Activities in Livers of APAP-Treated Mice

**Figure 8:**
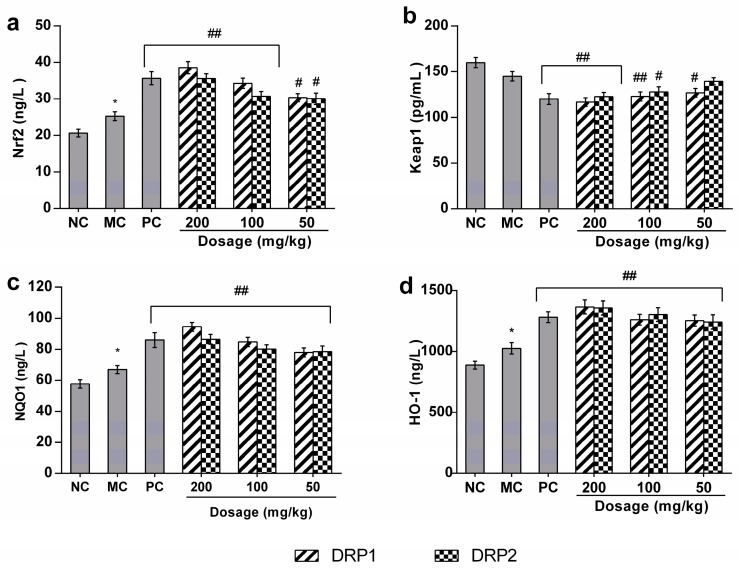
Effects of DRP1 and DRP2 on liver Nrf2, Keap1, NQO1 and HO-1 levels. (**a**), Nrf2 (**b**), Keap1 (**c**), NQO1 (**d**), HO-1. The values presented are the mean ± S.E.M. (*n* = 10 in each group). * *p* < 0.05 compared with the NC group; # *p* < 0.05, ## *p* < 0.01 compared with the MC groups.

The antioxidant enzymes play an important role in maintaining the intracellular redox balance. As shown in Figure 8d–f, compared to the NC group, hepatic total superoxide dismutase (T-SOD), catalase (CAT) and glutathione peroxidase (GPx) activities were significantly decreased in APAP-treated mice by 57.2%, 43.4% and 33.1%, respectively (*p* < 0.01). However, treatment with DRP1 and DRP2 reversed the antioxidant enzyme activities in a dose-dependent manner (*p* < 0.01).

#### 2.3.5. Polysaccharide Fractions -Mediated Protective Action Involves the Nrf2-Keap1 Pathway

The accumulated evidence showed that Nrf2 is a basic leucine zipper redox-sensitive transcriptional factor that plays a center role in ARE-mediated induction of phase II detoxifying and antioxidant enzymes. To further investigate the molecular mechanism of oxidative stress in mouse liver, we measured the expression levels of Nrf2, Keap1, NQO1, and HO-1 (Figure 8). The protein expression levels of Nrf2, HO-1 and NQO1 were increased in mice treated with APAP alone as compared with the the NC group, possibly due to a compensational response to the oxidative stress induced by APAP. Nevertheless, compared with MC group, treatment with DRP1 and DRP2 further increased the expression levels of Nrf2, HO-1 and NQO1 in mice liver.

As shown in Figure 8b, no significant differences in Keap1 protein levels were observed between the MC group and NC group, but compared with the MC group, the DRP1 and DRP2 treatment groups exhibited a decreased level of Keap1 expression.

## 3. Discussion

Dandelion has a long history of both medicinal and food use in China. All parts of dandelion can be used as a medicinal herb, especially the root area which is rich in polysaccharides. The biological activities of polysaccharides are heavily dependent on numerous factors including chemical components, molecular weight, glycosidic bonds, even the extraction and isolation methods [28]. In the present study, the crude DRP was obtained by water extraction, and separated through DEAE-52 and Sephacryl^TM^ S-200 column chromatography. The results demonstrated that the two isolated polysaccharides DRP1 and DRP2, with molecular weights of 5695 and 8882 Da, respectively were successfully isolated from the crude DRP. Monosaccharide analysis revealed that DRP1 was composed of glucose, galactose and arabinose, while DRP2 was composed of rhamnose, glucuronic acid, glucose, galactose and arabinose. The two polysaccharides belonged to the α-type polysaccharides. The backbone of DRP1 was mainly composed of (1→6)-linked-α-d-Glc and (1→3,4)-linked-α-D-Glc. DRP2 was mainly composed of (1→)-linked-α-d-Ara and (1→)-linked-α-d-Glc.

Serum alanine aminotransferase (ALT) and AST were used as biochemical indicators of liver injury [29], as studies show that increased serum ALT and AST activities are sensitive markers of liver injury [30,31] Therefore, to investigate the protective effects of DRP1 and DRP2 on APAP-induced liver injury, serum AST levels were firstly investigated. Our results demonstrated that APAP-induced liver injury, as reflected by increased levels of AST, was significantly blocked by DRP1 and DRP2 treatment. Histopathological analysis of liver tissues indicated significantly reduced areas of necrosis in mice after treatment with DRP1 and DRP2 compared with the APAP-alone group. These results indicated the protective effects of DRP1 and DRP2 against APAP-induced liver injury.

Studies showed that oxidative stress is the main factor that causes liver injury [32,33]. A major defense mechanism for prevention and treatment of liver damage comprises reducing the production of ROS by raising the levels of antioxidant enzymes, such as SOD, CAT and GPx. MDA, one of the most frequently used indicators of lipid peroxidation, was also used to assess the oxidative stress [34]. In the present study, DRP1 and DRP2 treatment increased GSH, GPx, T-SOD, CAT levels and decreased ROS, MDA levels, suggesting that DRP1 and DRP2 could significantly attenuate the oxidative stress induced by APAP, so the hepatoprotective effect of DRP1 and DRP2 might be associated with their anti-oxidative activity.

The induction of antioxidant enzyme genes is regulated by several cell signaling pathways and transcription factors [35]. Transcription factor Nrf2 is a master regulator of the cellular antioxidant response through the Nrf2-Keap1 signaling pathway [36]. In particular, Nrf2 has been reported to play a key role in protecting against APAP-induced liver injury, since Nrf2 knockout mice were more susceptible to APAP-induced liver injury [37,38].

Upon stimulation by inducers, Nrf2 dissocitates from Keap1 and translocates into the nucleus where it dimerizes with some cofactors and binds to ARE. This will lead to activation of a battery of highly specialized proteins, including NQO1, glutathione S-transferase (GST) and HO-1 that efficiently protect tissues and organs from various forms of toxicants [39]. To investigate the antioxidant mechanism of polysaccharide fractions, the effects of polysaccharide fractions on Nrf2, Keap1, NQO1 and HO-1 expression were detected. Our results showed that DRP1 and DRP2 could further enhance the protein expression of Nrf2, NQO1 and HO-1, decrease the protein expression of Keap1. These results suggested the antioxidant mechanism of polysaccharide fractions was through the Nrf2-Keap1 signaling pathway.

## 4. Materials and Methods

### 4.1. Materials and Reagents

Dandelion root was purchased from a local market in Jilin, China. DEAE-52 cellulose was obtained from Whatman (Balston, UK). Sephacryl^TM^ S-200 was purchased from GE Healthcare Bio-Sciences AB (Uppsala, Sweden). 1-Phenyl-3-methyl-5-pyrazolone (PMP) was from Xiya Chemical Industry Co., Ltd (Shandong, China). L-Rhamnose, D-glucose and D-galactose were from Beijing Zhongke Biochemical Technology Co., Ltd. (Beijing, China). D-Mannose, L-arabinose and D-glucuronic acid were from the National Institutes for Food and Drug Control (Beijing, China). Dextran standards (4320, 12,600, 60,600, 110,000 and 289,000 Da) were purchased from Shanghai Macklin Biochemical Technology Co., Ltd (Shanghai, China). APAP was obtained from Sigma Chemical Co. (St. Louis, MO, USA). Aspartate aminotransferases (AST), malondialdehyde (MDA), glutathione (GSH), glutathione peroxidase (GPx), total superoxide dismutase (T-SOD) and catalase (CAT) assay kits were all obtained from Nanjing Jiancheng Bioengineering Institute (Nanjing, China). Reactive oxygen species (ROS), Nrf2, Keap1, NQO1, HO-1 ELISA assay Kits were purchased from Nanjing Jin Yibai Biological Science and Technology Co., Ltd. (Nanjing, China).

### 4.2. Extraction of Crude Polysaccharide

Dandelion root was washed by distilled water and dried at 70 °C. The dry Dandelion Radix was ground into a powder. Then the powder was treated with ethanol (95%, *v*/*v*) in a Soxhlet system at 70 °C for 4 h to remove the majority of the monosaccharides, pigments and fats. The residue was then separated by filtration and dried at 40 °C in a hot air oven to obtain the pretreated dry sample powder. Then the pretreated dry powder (50.0 g) was immersed with distilled water (1200 mL) for 4 h at 100 °C. The solution was collected by centrifugation (4000 rpm, 15 min), and the residue was extracted repeatedly twice. All supernatants were combined and concentrated to 100 mL volume. Subsequently, anhydrous ethanol was added to the concentrated supernatants with constant stirring to achieve a final concentration of 80% ethanol and kept at 4 °C overnight. The precipitate was separated and dissolved in distilled water, then 1/4 volum Sevage solution was added. The mixed solution was placed in table concentrator and shaken for 30 min. After being static 30 min, supernatants were collected. The operation was repeated three times. Subsequently, the supernatants were extensively dialyzed (3.5 kDa Mw cut-off) against distilled water for two successive days. Then the solution was concentrated and freeze dried to obtain the crude DRP.

### 4.3. Separation and Purification of DRP

The crude DRP was re-dissolved in distilled water, and then purified by DEAE-52 and Sephacryl^TM^ S-200 gel filtration chromatography according to the reported method [40]. The crude DRP solution (10 mL, 10 mg/mL) was applied to a column (2.6 × 50 cm) of DEAE-52 cellulose which was then eluted in stepwise fashion with distilled water, 0.1, 0.3 and 0.5 M NaCl solutions at a flow rate of 1 mL/min. The fractions were collected at 5 min intervals with a fraction collector, and measured by the phenol-sulfuric acid method [41]. The major fractions were pooled, desalted, concentrated and further separated using Sephacryl^TM^ S-200 column (2.6 × 50 cm) which was eluted with distilled water at a flow rate of 0.5 mL/min. The fractions were collected and monitored under the same conditions as that used for the DE-52 cellulose column. Each fraction was collected, lyophilized and stored at 20 °C until use.

### 4.4. Determination of Contents of Carbohydrate and Protein

Total carbohydrate was determined by phenol-sulfuric acid method using glucose as the standard [42]. The protein content of purified polysaccharide was determined according to Bradford method [43]. All the data were detected by a UV-2450 instrument (Shimadzu, Tokyo, Japan). The polysaccharides were dissolved to 1 mg/mL solution and scanned from 200 to 400 nm with an ultraviolet spectrophotometer.

### 4.5. Infrared Spectroscopy Analysis

In this experiment, 2 mg of polysaccharide fractions were ground with 400 mg of dried potassium bromide power. The resulting powder was transferred to a compression mold and pressed under vacuum into tablets. The absorption spectra of the tablets were measured with a FT-IR spectrometer (Tensor 27, Bruker, Karlsruhe, Germany) from 4000 to 400 cm^−1^ according to the reported method [44]. Karlsruhe

### 4.6. Monosaccharide Composition Analysis

The polysaccharide samples (5.0 mg) were hydrolyzed for 8 h at 110 °C with trifluoroacetic acid (TFA, 4 mL, 4 M) in a sealed test tube. The PMP derivatization of monosaccharides was carried out following the reported method with some modifications [45]. Briefly, hydrolyzed polysaccharide sample or monosaccharide standard solution (200 μL) was mixed with 0.3 mol/L sodium hydroxide (240 μL). Then, the mixture was added with 0.5 mol/L methanolic solution of PMP (240 μL) and mixed thoroughly by a vortex mixer. The whole mixture was kept at 70 °C for 2 h in a thermostat water bath. The mixture was neutralized with 0.3 mol/L hydrochloric acid (240 μL) after being cooled to room temperature. Then, chloroform (1 mL) was added to the mixture which was shaken vigorously. The chloroform layer was discarded and the extraction process was repeated three times. The aqueous layer was filtered through a 0.45 μm filter for HPLC analysis. The derivatization procedure of both hydrolysate and monosaccharide standard samples must be carried out under the same condition. Monosaccharide composition was measured by HPLC (LC-2010A, Shimadzu, Tokyo, Japan) coupled to an UV-Vis detector and a BDS HYPERSIL C_18_ HPLC column (4.6 mm × 250 mm, 5 μm, Thermo Scientific, Sunnyvale, CA, USA) maintained at 40 °C. The column was eluted with an isocratic mobile phase consisting of acetonitrile and 0.05 M phosphate buffer (pH 6.8, 16:84, *v*/*v*) at 1.0 mL/min.

### 4.7. Molecular Weight Determination

The molecular weight of the polysaccharides was characterized by HPGPC according to the reported method with slight modifications [41]. Briefly, polysaccharide samples (25 mg) were dissolved in distilled water (5 mL), filtered through a 0.45 μm membrance, and applied to a Ultrahydrogel^TM^ Linear gel-filtration chromatography column (7.8 × 300 mm). Twenty μL of sample solution was injected in each run, with deionized water as the mobile phase at a flow rate of 0.8 mL/min. The column was calibrated with Dextran standards of known molecular weight (4320, 12,600, 60,600, 110,000, 289,000 Da). The molecular weight of polysaccharides was estimated by reference to the calibration curve made above.

### 4.8. Methylation and GC-MS Analysis

Methylation analysis was conducted using the method of Ciucanu and Kerek with some modifications [46]. Polysaccharide samples (5 mg) were dried overnight at 40 °C in a vacuum oven and dissolved in DMSO (0.5 mL) by sonication at room temperature. Methyl iodide (0.3 mL) followed by finely powdered dry sodium hydroxide (20 mg) were added to a stirred solution of the polysaccharide dissolved in DMSO under nitrogen at room temperature. Then, the mixture was stirred for two hours. The methylated polysaccharides were extracted with dichloromethane. The dichloromethane phase was washed three times with 1 mL of water and dried with a stream of nitrogen. The methylated polysaccharide was reduced by NaBH_4_ and then converted into partially methylated alditol acetates (PMAA) and analyzed by GC-MS (Agilent 7890/5975C, Agilent Technologies, Palo Alto, CA, USA) equipped with a HP-5 capillary column (30 m × 0.25 mm i.d., 0.25 μm film thickness).

### 4.9. Animals and Experimental Design

Male ICR mice (25–30 g) were purchased from the Qinglongshan Experimental Animal Breeding Farm (Nanjing, China). The mice were housed in cages under controlled conditions of 12 h light/dark cycles at 22 ± 2 °C and 50–60% humidity with free access to water and standard food. All procedures were conducted according to the institutional animal care guidelines and approved by the Institutional Ethics Committee.

After acclimatization to the laboratory conditions, the mice were randomly divided into six dosage groups (10 animals in each group) including DRP1 groups (200, 100, 50 mg/kg) and DRP2 groups (200, 100, 50 mg/kg), as well as three control groups including NC group, MC group and PC group. For the first time, the mice in NC groups were treated with physiological saline by intraperitoneal injection. And the other five dosage groups were treated with APAP at a dose of 400 mg/kg [47]. After 6 h, the intraperitoneal injection of polysaccharides in dosage groups were processed three times daily, using physiological saline in NC and MC groups, and *N*-acetylcysteine (120 mg/kg) in PC groups as control. The whole treatment was lasted for two days.

Animals were then killed 4 h after the last administration of polysaccharide fractions and blood samples were drawn by excising eyeball. Liver tissues were harvested, and a portion of the liver was fixed in 10% buffered formalin for histological assessment and the remaining tissues were stored at −80 °C for further use.

### 4.10. Histological Evaluations

Liver tissues were immediately immersed in neutral buffered formalin, embedded in paraffin, cut into sections. And then they were stained with hematoxylin and eosin (H&E) following a standard protocol. H&E-stained liver sections were used to evaluate liver damage using a light microscopy.

### 4.11. Serum Enzyme Assay

Blood samples were harvested immediately in centrifuge tube. After 2 h, the blood samples were centrifuged at 3500 rpm for 10 min to afford the serums required. The AST in serum was measured with a commercial reagent kit according to the manufacturer’s protocol.

### 4.12. MDA, ROS, GSH, GPx, T-SOD, and CAT Content Assay

The liver tissue was weighed and homogenized (1:9, g/mL) in phosphate buffer solutions (PBS, 0.2 M, pH 7.4). The homogenates were centrifuged (3000 rpm, 4 °C) for 20 min, and the supernatants were collected for further analysis. MDA, GSH, GPx, T-SOD and CAT activities in liver tissues were determined using relevant test kits according to the manufacturer’s instructions. Liver ROS content was measured with the ELISA assay kit according to the manufacturer’s instruction.

### 4.13. Determination of Nrf2, Keap1, NQO1 and HO-1

The liver tissue was weighed and homogenized (1:9, g/mL) in phosphate buffer solution (PBS, 0.2 M, pH 7.4). The homogenates were centrifuged (3000 rpm, 4 °C) for 20 min, and the supernatants were collected for further analysis. The levels of Nrf2, Keap1, NQO1 and HO-1 in liver tissues were tested using ELISA assay kits according to the manufacturer’s instructions.

### 4.14. Statistical Analysis

Data were statistically analyzed, using the GraphPad Prism 6.0 software (GraphPad Software Inc., San Diego, CA, USA), by one-way ANOVA. Significant differences between two means were determined by Tukey’s test. *p* < 0.05 or *p* < 0.01 were regard as significant.

## 5. Conclusions

In the present study, two purified water-soluble polysaccharide fractions, named DRP1 and DRP2, were separated and purified from Dandelion Radix, by DEAE-52 and Sephacryl^TM^ S-200 chromatography. Their preliminary structural characteristics and hepatoprotective effect were firstly evaluated and compared. Experimental results showed that DRP1 was mainly composed of glucose, galactose and arabinose, while DRP2 was mainly composed of rhamnose, glucuronic acid, glucose, galactose and arabinose. The average molecular weights of DRP1 and DRP2 were determined to be 5695 Da and 8882 Da, respectively. There were characteristic infrared absorption peaks of polysaccharides in the FT-IR spectra of DRP1 and DRP2. The backbone of DRP1 was mainly composed of (1→6) linked-α-d-Glc and (1→3,4)-linked-α-d-Glc, and DRP2 was mainly composed of (1→)-linked-α-d-Ara and (1→)-linked-α-d-Glc. This study also demonstrated that DRP1 and DRP2 have potent protective effects against APAP-induced liver injury in mice liver. Here we demonstrate that DRP1 and DRP2 administration attenuated APAP-induced histopathologic changes. In addition, DRP1 and DRP2 attenuated APAP-induced hepatic oxidative damage by inhibiting MDA, ROS generation and increasing liver GSH, GPx, T-SOD, CAT levels. Furthermore, the molecular mechanisms of the antioxidant activity of DRP1 and DRP2 showed that DRP1 and DRP2 induce Nrf2 activation by decreasing the expression of its inhibitor protein Keap1, lead to the enhanced expression of downstream anti-oxidative proteins including NQO1 and HO-1, and finally preventing APAP-induced liver oxidative injury.

## Figures and Tables

**Table 1:** Methylation results of DRP1.

Methylated Sugars	Linkages	Molar Ratios	Maior Mass Fragments (*m/z*)
3,5-Me_2-_Ara	1,4-linked Ara	8.95	43, 88, 101, 118, 129, 145, 161, 178, 222
2,3,4,6-Me_4_-Gal	1-linked Gal	2.15	43, 87, 99, 102, 118, 129, 145, 172, 205
2,3,4-Me_3_-Glc	1,6-linked Glc	15.48	43, 87, 102, 116, 129, 144, 162, 189, 254
2,6-Me_2_-Glc	1,3,4-linked Glc	10.43	43, 98, 111, 127, 143, 157, 181, 217, 243
3,6-Me_2_-Glc	1,2,4-linked Glc	8.67	43, 88, 99, 113, 127, 130, 140, 169, 198
3,4-Me_2_-Glc	1,2,6-linked Glc	3.86	43, 85, 100, 118, 129, 190, 236

**Table 2:** Methylation results of DRP2.

Methylated Sugars	Linkages	Molar Ratios	Maior Mass Fragments (*m/z*)
2,3,5-Me_3-_Ara	1-linked Ara	4.96	43, 87, 102, 116, 129, 145, 178, 220
3,4-Me_2_-Rha	1,2-linked-Rha	0.34	43, 89, 100, 116, 130, 143, 190
2,4-Me_2_-Glc	1,3,6-linked Glc	1.71	43, 87, 100, 118, 127, 139, 169, 234
2,3,4,6-Me_4_-Glc	1-linked Glc	4.58	43, 87, 102, 116, 127, 145, 172, 205
3,6-Me_2_-Glc	1,2,4-linked Glc	1.37	43, 88, 99, 113, 130, 167, 190, 218, 233
2,4,6-Me_3_-Gal	1,3-linked Gal	0.52	43, 87, 101, 118, 129, 143, 161, 181, 234
3,4-Me_2_-Gal	1,2,6-linked Gal	2.15	43, 87, 100, 116, 127, 190, 232

Glucose-based sugar residues (including 1,3,6-linked Glc, 1-linked Glc and 1,2,4-linked Glc) were highly enriched in DRP2, which were substantially consistent with the results of monosaccharide composition analysis mentioned above.
